# Annual Address

**Published:** 1873-04

**Authors:** J. T. Johnson

**Affiliations:** Atlanta, Georgia


### ANNUAL ADDRESS,

*Delivered Before the Georgia Medical Association, Atlanta, Georgia, April* 10, 1873.

BY J. T. JOHNSON, M.D., ATLANTA, GEORGIA.

*Gentlemen of the Georgia Medical Association:*—"Man is a dupable animal; quacks in medicine, quacks in religion and quacks in politics know this, and take advantage of the knowledge. There is scarcely any one who may not, like a trout, be taken by tickling." This quotation suggests "The Science of Humbuggery."

It may be objected that this expression is a paradox; that it is absurd to speak of the science of that which is notoriously unscientific. But the science of anything may be defined as the knowledge of the laws that govern it. True, in one sense, humbuggery is not governed by laws. But its growth and its life depend on the knowledge of the principles (or for the want of them, it may be suggested,) that control man and regulate society. Its most successful practice, and its most successful resistance, depend on the appreciation of the circumstances that give rise to it and lend it strength. It depends on the undiscerning judgment—or, more plainly, the ignorance—of mankind on one part, and its grasping, heartless cupidity on the other.

But, yet, we may console ourselves with the thought that our avocation is not the only one that suffers from this monster's insidious presence. We find the slime of its poisoned trail, and the imprint of its envenomed tooth, in every class of men, and in every walk of life. It rears its brazen face, and sounds its pompous voice, in every clime and in every tongue. Society is hampered by it; commerce gloats upon it; politics fester with it; the professions are burdened by it; and let us blush as we think of it, even religion is tinctured with it.

But we haven't time for all this. Let us consider it as found around us—among us. Let us lift the veil that hides the hideousness of its skeleton; let us examine the gaudy dress with which it catches the wondering financial eye.

There is in every one a love of being deceived—an unexpressed fondness for the rascality that titillates with one hand, and feels the depth of the pocket with the other. Men and women possess a capacity for being gulled, and that capacity often of incredible extent. Children have often been promised the first money floating down the river on a mill-stone. Grown-up children are as readily taken by bait not more alluring, and not less thin.

We find, too, deep down in the breast of many who will never acknowledge it, a latent, undeveloped love of superstition. True, they may laugh at the possibility of ghosts or hobgoblins, but they will seek to rob circumstances of their commonplace reality, and invest them with a supernatural tinge.

And this suggests the humbuggery of the day, the grand climax of dissolving wonders, Spiritualism. True, it does not fall altogether under the head of medical humbuggery, for this is but one of the many assumed powers, but one of the thousand domains, over which it claims to sweep with tread omniscient and omnipotent.

For all this, I should think it unworthy of notice here, but it has become so presumptuous that even ministers of the gospel have thought it not unbecoming to descend from the pulpit they should honor, dragging, though unintentionally, the garments of their Master in the dust, and wrangle in the market places with the blatant blasphemers of His word.

The attempt of these men to apply their wondrous powers to the healing of the sick is almost too weak to be dignified as humbuggery. True medical science can never suffer injury at the hands of these latter-day prophets, whose brains are become the disordered workshop in which the Devil finds tools for his work of destruction, and whose creed is but the sugar-coated pill of infidelity.

Their medical treatment consists of a confused mingling of spiritualism, mesmerism, laying on of hands—in short, anything invisible or intangible that is calculated to appeal to the love of novelty or superstition as existing among the less-informed of a community. There exists among these people an ill-defined idea that words flash along wires, thoughts through brain, and pain along nerves, by the same unseen force. You will be told by them that all pain—whether the merest nervous twinge of the hypochondriac, or the hopeless pangs of the tortured sufferer, depend on a deficiency of this power—a power only physical, not vital—and that for the cure nothing is necessary except that some favored medium with a credulous brain, a heart of benevolence, and a body loaded with domesticated curative lightning, allow some of this holiness to depart from him and enter the writhing mortal before him.

As touching on the influence of mind over body, I may do well to give a brief account of the once celebrated "Perkin's Tractors" that wrought such astonishing cures in the beginning of the century, in England. These tractors were pieces of metal that were to be drawn over diseased or painful portions of the body. Their effect was attempted to be accounted for by some supposed galvanic, electric or magnetic power exerted by the metals of which the instruments were composed. It was determined at some of the hospitals to put them to the test. Some wooden tractors were prepared and so painted as to resemble the mysterious original ones. A number of the hospital patients were selected and submitted to what they supposed to be the treatment by the genuine tractors. These patients labored under various diseases of a chronic character, including gout and rheumatism. Upon the slightest strokes of this painted wood, all declared themselves relieved. Some improved instantly so much in their walking as to delight in giving evidence of the benefit they had received. They complained of wonderful sensations produced by the contact of the wood. Similar experiments were made at several of the hospitals. The fame of the cures spread to such an extent that more patients crowded for relief than there was time to devote to them. Men who had been unable to raise their arms could now with ease carry coals or other objects of considerable weight. This test of humbuggery seems incredible but for having been witnessed by many observers, and well authenticated. "Such cases go far to explain how miraculous cures are to be ascribed to empirical remedies, many of which are composed of substances most inert in their nature. It is the confidence of the quack and the hope of the patient that work the cure. Disease is well known to depress the power of the understanding as well as the vigor of the muscular system, and will deprave the judgment, not less than the digestion. A sick person is extremely credulous about the object of his hopes and his fears. Whoever promises him hope, may easily obtain, for the time being, his confidence; and he can thus easily become the dupe of quacks and ignorant pretenders."

Professor Woodhouse has given an account which helps to show what singular effects can be produced if the person be previously prepared for the production of wonders.

When nitrous oxide gas first excited attention, several of his friends were exceedingly anxious to inhale it. Instead of the gas, the Professor administered to them several quarts of atmospheric air from .the apparatus they had seen used for the gas. So thoroughly were they impressed, that a train of symptoms followed consisting of quickness of the pulse, dizziness, vertigo, tinnitus aurium, difficulty of breathing, anxiety about the chest, sensations of swinging, faintness, weakness, and nausea lasting six or eight hours—all from inhaling common air under an excited imagination. Under such circumstances, if one really suffer, and even intensely, his mind may be so wrought upon by the process as to cause him to lose or forget his ills.

The instance is often related of the condemned criminal who was bled to death without losing one drop of blood. Having been told that he must so suffer, he was blind-folded and deceived by letting fall a stream of water upon his arm. The poor fellow actually died of deception.

This power of deception over a person, through his fears and hopes, by what he sees and what he hears, is never credited by the victim; nor can it be imagined by any one who has never given the subject special attention. It is applicable to the robust in health or the invalid, old or young, those of otherwise sound judgment, and those of weaker brain.

The patient will place his confidence in any one whom chance may throw in his way. It may be some honest and capable man whom he has known from his childhood; or, more likely, in some straggling imposter, who is himself over-confident because he has not the knowledge to appreciate danger, or the brain to generate doubt.

Upon diseases severe, even incurable, the influence of confidence, for the time, is often marked. All of us have noticed this effect in epilepsy. This is a real, a terribly real disease—one that does not depend on a whim of the patient's imagination—and yet there is often an improvement, or apparent cure, upon the change of prescription or physician.

The hysterical and the hypochondriacal suffer their hundred ills, springing from the acuteness of a disordered imagination; yet, to them, ridicule them as we will, they are more terrible than realities. Was one of their whims ever laughed out of existence, or cured by disbelief? But by deception, often. As they are the subject of most absurd delusions, so they readily become the victims of most worthless quacks.

But though we may be amused by the imposter's successful play upon the changing panorama of such imaginary woes, far differently must we feel when we see the hopeless victim of an unrelenting disease yield himself to flattering and false hopes, only that the love of gain of some grasping wretch may be gratified. Here is a picture from the greatest of novelists: There is a disease which so prepares its victim, as it were, for death; which so refines it of its grosser aspect, and throws around familiar looks unearthly indications of the coming change; a dread disease, in which the struggle between soul and body is so gradual, quiet and solemn, and the result so sure, that day by day, and grain by grain, the mortal part wastes and withers away, so that the spirit grows light and sanguine with its lightening load, and, feeling immortality at hand, deems it but a new term of mortal life; a disease in which death and life are so strangely blended, that death takes the glow and hue of life, and life the gaunt and grisly form of death; a disease which medicine never cured, wealth never warded off, or poverty could boast exemption from; which sometimes moves in giant strides, and sometimes at a tardy sluggish place, but, slow or quick, is ever sure and certain."

To see one thus doomed, sinking through a living death, holding on to what is left of life with all his feeble strength, hoping against hope, struggling against despair, creeping from place to place, and clime to clime, dodging yet a little longer the grasp of the still inevitable monster, is sad indeed. But sadder still is it to see him place his last earthly hope on the reckless promise made by some soulless quack that he may sell for a few paltry cents his bottle of syrup warranted to cure consumption.

But to return to the effect of imagination: How many are before me who, when first conning their text books, were afflicted with every disease they studied? You would read of heart disease; there was the picture before you, and though not in reality possessed of one of its features, yet you were firmly persuaded that ere long you must drop from its sudden inroads. And so you would still think, unless the next day's chapter doomed you a lingering victim of tubercle.

One of the ready tools of humbuggery, its mighty engine with which it attempts to batter down the strongholds of truth and science, is advertising. Printer's ink draws better than doctor's plasters. It is a maxim with those who pursue this business, that so much advertising brings so much reputation, and so much reputation so much money. Anybody may establish any medicine as "good" for any disease, if he will advertise it judiciously. Should a disease recover in its own good time, the cure is of course attributed to the pills with which it was peppered. The advertiser well knows that no failure will retard the sale, (for it is not made public,) and that every apparent cure gives it reputation.

The advertiser easily persuades his reader that his "blood is impure;" that he must "correct the secretions;" that he must "regulate his liver." What seductive phrases are these? How glibly they roll from the tongue? What nice handles for the tongue to seize upon? What elastic terms to comprise so much unmanageable ignorance?

The blood is but a seething mass of corruption, a raging torrent of impurities, ever tossing to the surface as proof of the putrid mass within, its pimples and its blotches. Let him but drop into it a little of the advertiser's magic balm, and all is purity again.

"Correct the secretions!" Neither the advertiser or the victimized knows what they are or what they should be; and if they did, they do not know how to correct them; and if they knew how, have nothing with which they can accomplish it.

The liver! Here we have a fountain forever flowing with advertisers' diseases; the hub around which revolve all the diseases that flesh is heir to; the therapeutical scapegoat on which are piled all the sins of ignorance. It is the mischief-maker of the body; the organ whose chief function is to be forever out of order. Possessed of a name more generally known than any other viscus, it must always suffer the penalty of its notoriety. It is blown upon both hot and cold: if accused of torpidity, one remedy; if over active, the same.

Poor, innocent liver! Thy anatomy a blunder, and thy function a mistake! Doomed forever to float in a halo of biliousness, and to be forever plied with pills and powders! Oh, the ignorance thy existence has fostered, and the crimes done in thy name!

This has suggested another mighty weapon of humbuggery—certificates. You can procure certificates of any kind, and in any number, for anything. You may beg them, buy them, or make them. You may get them honestly or you may deceive the certifier. But they are written by him who has his axe to grind, and not by him who turns the stone.

The quack well knows that one cure, or one recovery under treatment, or even one invented cure, well advertised, will bring more patients than hundreds of cures honestly and unostentatiously done. No matter how simple may be a disease or an operation, advertise it, and you at once confer upon it a miraculous and enchanting hue.

These newspaper doctors have powerful allies in the reverends, so-called. A toll-gate keeper who hailed from the land of wit and bogs, was told by a passer to whom he applied for toll fee, "I am a minister." "All right," says Pat, "pass on." "But," says he, "how do you know that I am really a preacher?" "Sure," says Pat, "if you are a preacher, you wouldn't lie about it, would you?" Many who read the certificates have a philosophy no deeper than this. But these reverends are not always empty prefixes, for there are some who are too ready to make a display of their signatures about matters of which they are ignorant; having filled pulpits with their eloquence, they fill columns with their certificates. Let us hope that growing observation will soon enlist them on the side of decency and respectability.

There is another division of this subject that I hope I may approach with due respect. I allude to humbuggery in the profession. We may ask here, What constitutes a physician? Is it the mere possession of a legal diploma? Is it an observance of the Code of Ethics? Yes, it is this, but more than this. It requires an honest capacity to appreciate the principles of the science that have been deduced from study and observation. It requires a moral strength that will resist mercenary seductions, and adhere to the obligations assumed when enlisting in the ranks of the profession.

There are those who honestly criticise some of the provisions of the Code of Ethics—and, yet, observe them. I do not advocate it as its blinded partisan. No instrument of its length has ever yet fallen perfect from human brain—and never will. There may be an honest desire and an actual necessity for its amendment. But, still, when I hear a doctor continually grumbling that it is for the good of the favored few, and that it works to the detriment of the many younger or lesser lights, I then look beneath the surface for the secret cause of his charges. I suspect that he is trying to twist a code into the shape of his practice. I am not surprised if he is stricken ere long with an *ism* or a *pathy*. I suspect that he is falling behind the age, and losing interest in the progress of the profession; that he is forgetting his text books, and is not learning anew from the journals; that he has adopted the profession from unworthy motives, and that there is creeping over him the foul atmosphere of policy and expediency; that he desires more the praises of the undiscerning rabble than the respect of the scientific physician; in short, that he has all the premonitory symptoms of professional demoralization.

What if the code is not the embodiment of wisdom, it is the criterion by which the respectable physician is recognized. It is the law of our profession, and each member should abide by it or repudiate it. If he will stab it, let him withdraw from the ranks and thrust as an open enemy, rather than give the assassin's blow under the guise of friendship.

True, there are those who attempt to ridicule this code. Rogues ridicule the law; skeptics ridicule the Scriptures; shall we, for that reason, become thieves and doubters? To gratify grumblers, shall we descend to their level? Shall we blot out the line of demarcation between the physican and the imposter who steals the title? Must we attempt no distinction between honest capacity and babbling ignorance? Shall every one who can induce his neighbor to call him "Doc" be admitted within the charmed circle?

True, the code has not eradicated all professional differences; true, it has not made an honest man of every doctor, nor a Solon of every fool. Some who claim the ægis of its protection would not live to the teachings of any code, moral or ethical. But before condemning the document, let us first try what virtue there may be in observing it.

As rigid as may be regarded some of its requirements, there is scope within its boundaries, and room in the play of its machinery, for a vast amount of professional floating. The very conditions of society and civilization that give rise to the more arrant quackery out of the profession, also encourage it within the ranks. They induce those underhand practices that no code can reach, and no law prevent. Within our ranks he that depends on intrigue or accident for success, rather than on more enduring merits, need never lack opportunities for the execution of his designs. They present at every turn of his work. There are those who walk habitually near the bounds of respectability, skillfully edging to keep on the safer side. Practice has discovered to them how great a looseness may be indulged in without bringing upon their necks the professional guillotine.

The ignorance of the patients in regard to themselves and their ailments is profound. In most instances, this ignorance may be made to run in any channel that one may desire. "*Post hoc, propter hoc*," is always their philosophy. The natural course of any disease, and its tendency to recovery of its own accord in most instances, is to them a sealed book. Believing that every disease must be cured, they naturally attribute every cure to the medicine given. The true physician well knows that in most instances of acute sickness, all that he can do is to allow the patient an opportunity to get well—he merely refraining from killing him while he gets well. Could this be understood, what an amount of quackery and undeserved praise must fall before it. If, indeed, the physician would always keep before him that "*post hoc*" is not "*propter hoc*," what volumes of false experience would be now unwritten.

To enumerate all the tricks of the trade would be useless, and neither profitable or agreeable. It is hard to learn that professional honesty is the best policy, especially when it must keep a man so poor. But what honesty is there in being honest for mere policy's sake?

It may be that we do not always defend a professional brother as valiantly as we should, when we hear him attacked for mishaps that we know he could not prevent. It is wrong to let him sink beneath an unjust accusation; but more wrong than this is it to decry his judgment, or call his treatment in question, when there are, from its nature, so many thousands of honest differences—often unimportant ones—in our practice. And this meanness can reach its lowest depth when cutting insinuations are made to do the work of slander more deeply than direct accusations. There are thousands of vulnerable points wherein may be thrust the foul shafts of professional envy; there are thousands of ways in which one may take to himself honors not rightfully his, and magnify himself and his skill at the expense of truth and honorable dealing; there are thousands of avenues that may be turned to lively account in the practice of respectable humbuggery.

Patients glory in having unusual or severe diseases. What more pleasure there can be in shuffling off this mortal coil to the tone of some rare exotic of a disease, than by the good old routes of pneumonia or bilious fever, I am unable to conceive. But it certainly is the case that patients are very fond of magnifying the dangers through which they have passed. They delight to tell to staring eyes and gaping mouths that they were let down "so near death's door as to hear the creaking of its hinges," and that it still closed between them and eternity. Why this should be so I cannot say; it is one of the innate human peculiarities. And he whose moral thermometer is about zero, or a little below, can take advantage of it for his own aggrandizement. What use is there in curing a patient of headache when you can as readily persuade him that he was well nigh dead with meningitis? What honor is there in curing headache? But curing meningitis requires a very special as well as a very valuable skill. Why cure him of sore throat, when he is so easily persuaded that you rescued him from the horrors of diphtheria?

Your patient knows nothing of the pathology of disease. He is a pathological fiddle on which you can scrape any tune you wish. He is always ready and anxious to believe that his internal anatomy is all shaken up, his viscera topsy-turvy, and his machinery badly tangled and out of order. Tell him so, if you like notoriety more than truth, and reap the reward of your dishonor.

There is a common idea that we must humbug to practice medicine. I do not know who is responsible for its dissemination, but I do know that we need not confirm it. It may not be humbuggery to give a bread pill. Perhaps it might be better for the patient if we would give them oftener. It is often necessary to give no remedy, and if your patient must "take something," why not do him up a crumb of comfort in a crumb of bread? But, lest some non-professional is disgusted at the possibility of having his devoted stomach so imposed on in times gone by these harmless frauds, I will add, that we rarely, if ever, resort to a remedy so useful as the bread pill.

What is the remedy for this small, low trickery within the profession? Neither law or ethics can reach it directly. You cannot entirely prevent it any more than you can stop little rascality in the non-professional world. But we can gradually and persistently inculcate the spirit as well as the letter of the ethics. Enforce it in your societies. See to it that the price of affiliation is a course of honorable dealing. No physician, unless a hardened criminal, professionally, will brave the shame of expulsion. As our country advances in refinement and education, our doctors will be more uniformly men of the better stamp. We may hastily say that the better people sometimes are taken in the toils of the most ignorant imposters. This may be true in isolated instances, but, as a rule, the more intelligent the communities, the more intelligent the doctors that live in them. Every public school though aimed afar off, is a shot leveled at this monster's head. Still enforce your ethics as well as you can, and in the course of years the whole lump will be leavened.

As to the prevention of the baneful quackery without the ranks of the profession, we are certainly unable, in the present condition of society, to accomplish it. The time is coming, though I fear yet far off, when this country shall have advanced much beyond its present status; there will be a demand for the physician, and not the uneducated quack. Then, there will be in our legislative halls men who can comprehend that the physician who has devoted his time and talent to the study of a science knows more of it than him whose capacity can scarcely master the alphabet.

But now let this body, if it will, representing the medical men of the State, present to our Legislature a paper upon any subject appertaining to the interest of the profession, or the health of the people, and what will be its fate? It will be laid on the table. 'Tis beyond their capacity, and they treat it with contempt. Yes, they will lay it on the table, and take up a bill to tax dogs, change county lines, or increase the fees of some justice of the peace. No, legislative quacks like medical quacks —and there is the secret of it.

Your Legislature will, time and again, enact careful laws to prevent some dishonest manufacturer from exploding the people with petro oil, or gunpowder, or nitro-glycerine; but the people must be allowed to blow up themselves or neighbors, whenever they choose to indulge in such an exhilarating luxury, with nostrums more dangerous far to the human family than all these inflammables combined.

How many bills do we see passed to allow certain persons to practice medicine! The representatives are indeed well prepared to pass upon the qualifications of applicants for the degree of Doctor of Medicine! The applicant having some admiring friend to sing his praises in the body, is dubbed with his title without, perhaps, one essential for the work he undertakes. The Legislature, fearing that the colleges are not of a capacity sufficient for grinding out doctors to supply the demand, thus supplement their power by this "easy method for beginners."

At this time we are cursed to an unusual degree with every form of quackery and humbuggery. We find it reaching out its arms in every avenue; it rolls from every press; it sweeps through every mail; and is emblazoned on every tree and fence board. We grimly stand on our professional dignity, and let ignorance and assumption run riot over us. We must stand with mute lips and folded hands, and hear defamed the principles we practice. Brainless fools flaunt in our very faces their flaming announcements and boasting lies. They publish to the world that we are old fogy fools, and not a voice is raised to defend the noble calling whose honor is in our keeping.

There is nothing that the true physician more desires than that the people were able to form the real estimate of all engaged in the practice of medicine; that they had the power that would strip empty assumption of its weapons, and grasping avarice of its arms. But from the nature of the science, from the ignorance of the public, for the lack of appreciation of cause and effect, the patient can not individually form an estimate of the merits of the adviser. Only the physician can judge of the ability of the physician. The physician's life is truly a hard one. There are trials and disappointments unnumbered, responsibilities overwhelming, services unappreciated, motives misconstrued, labors unrewarded, vigils unceasing. His life would spoil the patience of a Job, or the temper of a saint. But with all the hardships, there is nothing that so taxes his patience, so grinds his soul, as this lack of discrimination on the part of the people. From the heart's depths of every true physician goes the impatient yearning that fraud might be exposed in its iniquity, and ignorance in its emptiness. Still, through misappreciation and through calumny, let us go on with our mission. We know our rights, and let us dare maintain them. Let there be no spies within our lines and no enemies in our ranks. Let us inscribe on our banners science and progress; let fidelity and honesty be the unchanging countersign; let us move on, strong of truth and purpose, and full of the spirit of earth's noblest calling. Let us move on with steady front and solid ranks, heart with heart and shoulder to shoulder, ever clinging to the principles we cherish, always battling for the profession we love.

